# Integration of Seismic and Petrophysics to Characterize Reservoirs in “ALA” Oil Field, Niger Delta

**DOI:** 10.1155/2013/421720

**Published:** 2013-08-27

**Authors:** P. A. Alao, S. O. Olabode, S. A. Opeloye

**Affiliations:** Department of Applied Geology, Federal University of Technology Akure, P.M.B 704 Akure, Ondo State, Nigeria

## Abstract

In the exploration and production business, by far the largest component of geophysical spending is driven by the need to characterize (potential) reservoirs. The simple reason is that better reservoir characterization means higher success rates and fewer wells for reservoir exploitation. In this research work, seismic and well log data were integrated in characterizing the reservoirs on “ALA” field in Niger Delta. Three-dimensional seismic data was used to identify the faults and map the horizons. Petrophysical parameters and time-depth structure maps were obtained. Seismic attributes was also employed in characterizing the reservoirs. Seven hydrocarbon-bearing reservoirs with thickness ranging from 9.9 to 71.6 m were delineated. Structural maps of horizons in six wells containing hydrocarbon-bearing zones with tops and bottoms at range of −2,453 to −3,950 m were generated; this portrayed the trapping mechanism to be mainly fault-assisted anticlinal closures. The identified prospective zones have good porosity, permeability, and hydrocarbon saturation. The environments of deposition were identified from log shapes which indicate a transitional-to-deltaic depositional environment. In this research work, new prospects have been recommended for drilling and further research work. Geochemical and biostratigraphic studies should be done to better characterize the reservoirs and reliably interpret the depositional environments.

## 1. Introduction

Reservoir characterization has evolved through three generations—based originally in petrophysics, then on geologic analogs, and more recently on multidisciplinary integration. The petrophysical approach begun in the 1950s, where reservoir attributes were determined using logs, cores, and well tests. The second generation based principally on geologic analogs added the concept between wells. The analog technique improved interwell prediction, but identifying the right analog often proved difficult, especially in structurally and stratigraphic complex settings. Multidisciplinary approach, the third generation, attempts to integrate all available geologic, engineering, and geophysical data along with modern probabilistic and risk analysis techniques to produce a better reservoir model [1]. This third generation reservoir made significant use of 3D seismic amplitude anomalies and other complex trace attributes to define field development programs. This multidisciplinary approach was used in this research work.

## 2. Geologic Setting

**Figure 1:**
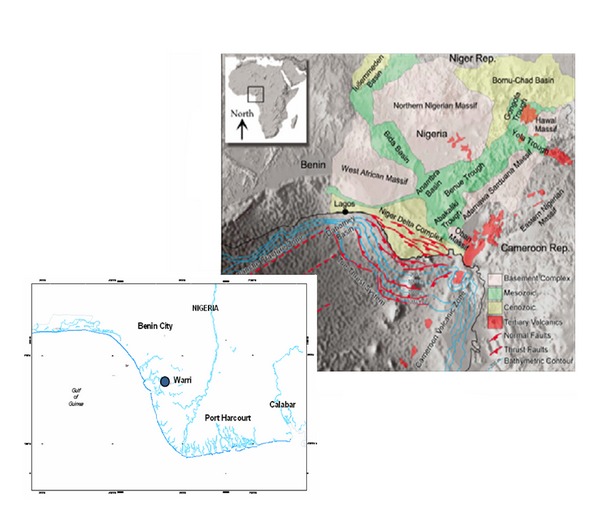
Location of “ALA” field in the Niger Delta.

The study area is located in the Cenozoic Niger Delta (Figure 1) which is situated at the intersection of the Benue Trough and the South Atlantic Ocean where a triple junction developed during the separation of the continents of South America and Africa in the late Jurassic [2]. Subsidence of the African continental margin and cooling of the newly created oceanic lithosphere followed this separation in early Cretaceous times. The Niger Delta started to evolve in early Tertiary times when clastic river input increased [3]. Generally, the delta prograded over the subsidizing continental-oceanic lithospheric transition zone and during the Oligocene spread onto oceanic crust of the Gulf of Guinea [4]. The weathering flanks of outcropping continental basement sourced the sediments through the Benue-Niger drainage basin. The delta has since Paleocene times prograded a distance of more than 250 km from the Benin and Calabar flanks to the present delta front [5]. Thickness of sediments in the Niger Delta averages 12 km covering a total area of about 140,000 km^2^. The stratigraphic sequence of the Niger Delta comprises three broad lithostratigraphic units: a continental shallow massive sand sequence—Benin Formation, a coastal marine sequence of alternating sands and shales—Agbada Formation, and a basal marine shale unit—Akata Formation. The Benin Formation is characterized by high sand percentage (70–100%) and forms the top layer of the Niger Delta depositional sequence. The Agbada Formation consists of alternating sand and shales representing sediments of the transitional environment. The sand percentage within the Agbada Formation varies from 30 to 70%, which results from the large number of depositional offlap cycles. The Akata Formation consists of clays and shales with minor sand intercalations. The sediments were deposited in prodelta environments. The sand percentage here is generally less than 30% [6]. 

## 3. Methodology

Data set used in this study includes 3D seismic and 6 well logs provided by Chevron Nigeria Limited (CNL). The structure of the field of study was interpreted using 3D seismic data which was integrated with well logs. The inline and crossline numbers range from 5800 to 6200 and from 1480 to 1700, respectively. The reflection quality of the data is very good; faults and stratigraphic picks for horizons are easily recognizable. The wells (ALA 01, ALA 02, ALA 03ST, ALA 04, ALA 05, and ALA 06I) penetrated depths of −13,019.00 ft (3968.19 m), −12996.00 ft (3961.18 m), −13000.69 ft (3962.61 m), −11,541.5 ft (3517.85 m), −11674.50 ft (3558.38 m), and −13088.50 ft (−3989.37 m). In order to increase the accuracy of subsurface imaging, generate maps and well log cross-sections, geophysical softwares like SMT Kingdom Suite, Schlumberger's Petrel, and Senergy's Interactive Petrophysics were used to produce a comprehensive geophysical and geological evaluation of the study area. The acoustic velocities from the sonic logs were multiplied by density log to compute new acoustic impedance (AI) log. This impedance was converted to reflectivity, which was then converted from depth to time using an appropriate wavelet to produce a synthetic seismogram for well ALA 04 (Figure 3) to tie the wells to seismic.

**Figure 2:**
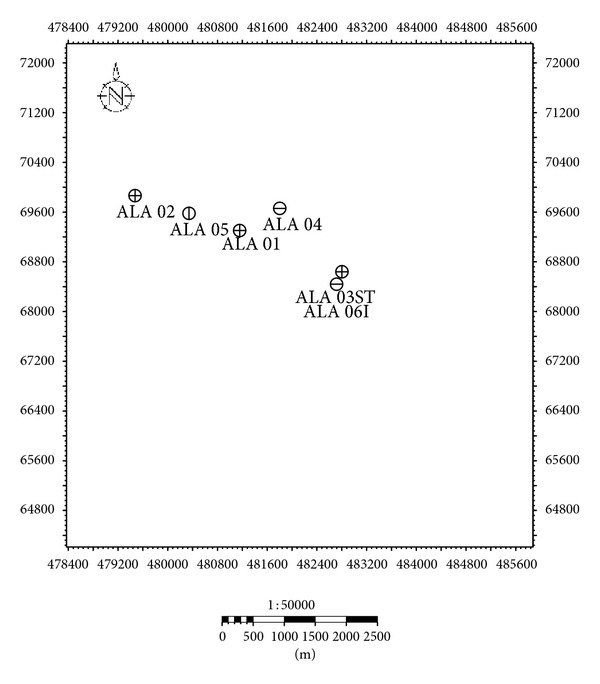
Basemap showing location of the wells.

**Figure 3:**
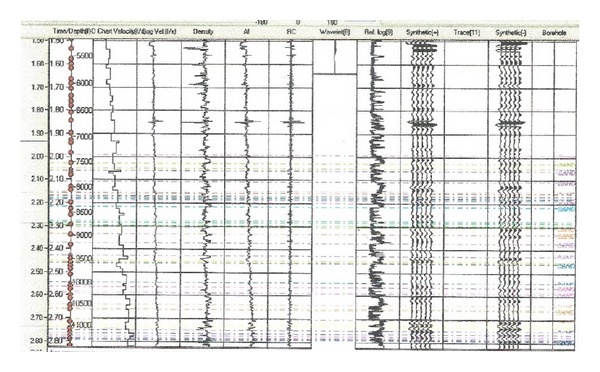
Synthetic seismogram of well ALA 04.

**Figure 4:**
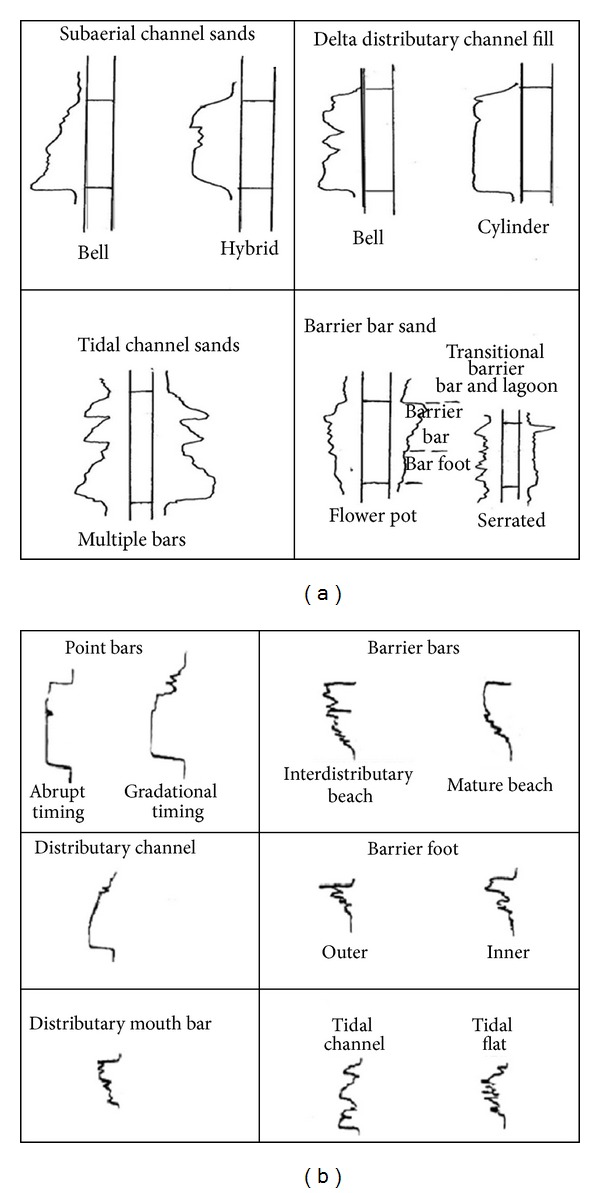
Gamma ray and resistivity log shapes suggestive of depositional environment: (a) After Busch, 1975 [7]; (b) Schlumberger, 1985 [8].

The wells used for this study were plotted as they appeared on the base map (Figure 2), that is, from west to east. The plot revealed that the sands reduced in thickness going from west to east. The direction of deposition of the sands was thus inferred to go from proximal (west) to distal (east). Sand units of interest were carefully picked and correlated across the wells to give an idea of the continuity of the reservoirs at different depths across the whole survey area. Petrophysical parameters obtained in the course of study includes volume of shale, porosity (effective and total), net-to-gross ratio, water and hydrocarbon saturation (moveable and residual), irreducible water saturation, bulk volume of water, permeability, and fluid discrimination. The fluid types were discriminated using the neutron-density log. In order to properly characterize the reservoir sands delineated and correlated across the studied wells, a plot of *S*_*w*_ versus Φ (i.e., Buckles plot) was made to determine whether or not the sands are at irreducible water saturation. The depositional environments were inferred from gamma and resistivity log motifs after Busch and Schlumberger models (Figures 4(a) and 4(b)).

## 4. Results and Discussion

**Figure 5:**
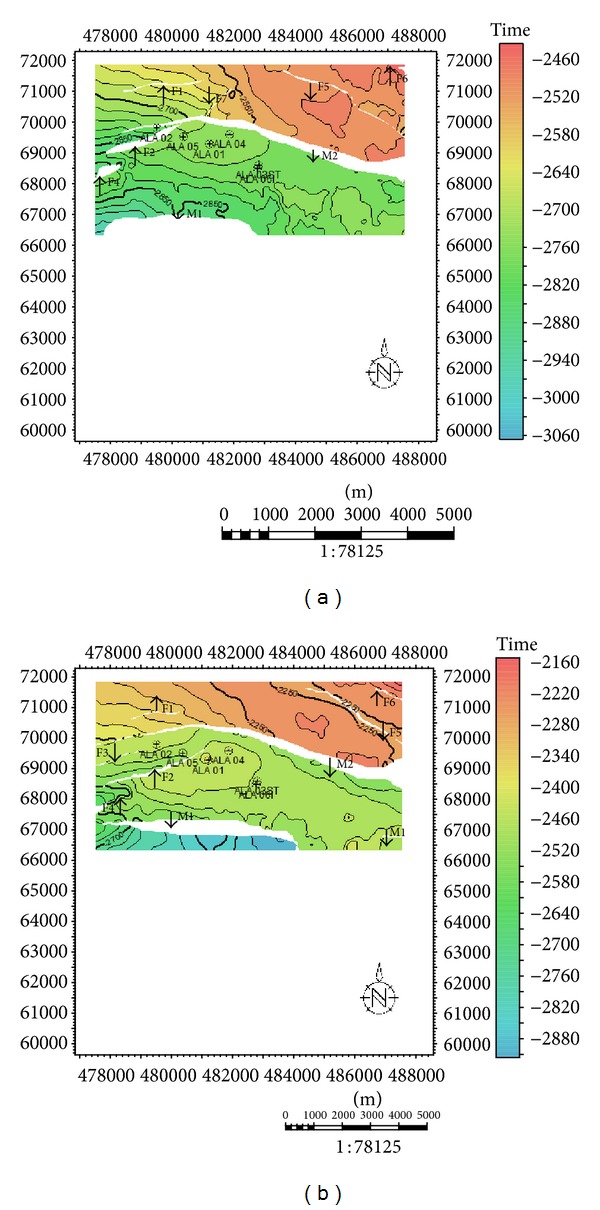
Time structure map for reservoirs A and D. (a) Time structure map for reservoir A (b) Time structure map for reservoir D.

**Figure 6:**
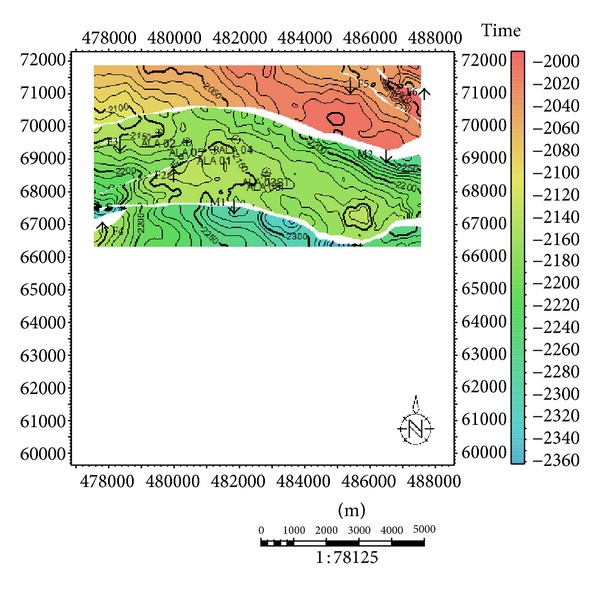
Time structure map for reservoir G.

Time structural contour maps were produced for three horizons defined on top of sand bodies, namely, Hor A, Hor D, and Hor G. The maps generated revealed two major faults (M1 and M2) which are growth faults and are very extensive (Figures 5 and 6). The major faults trend NE-SW and dip south terminating at the northwest flank of the field. The major faults show a subparallel relationship. The field is dissected by several crestal synthetic and antithetic faults. Most northerly minor faults are synthetic to the major fault M2; those at the central parts are antithetic. These intrafield small-displacement faults are of varying lengths and most run almost parallel to the north bounding major structure building fault. The field structure generally strikes almost perpendicular to the major structure building fault. The structure climbs to the east where the highest points in most of the reservoirs encountered in all the wells drilled on the field are recorded.

**Figure 7:**
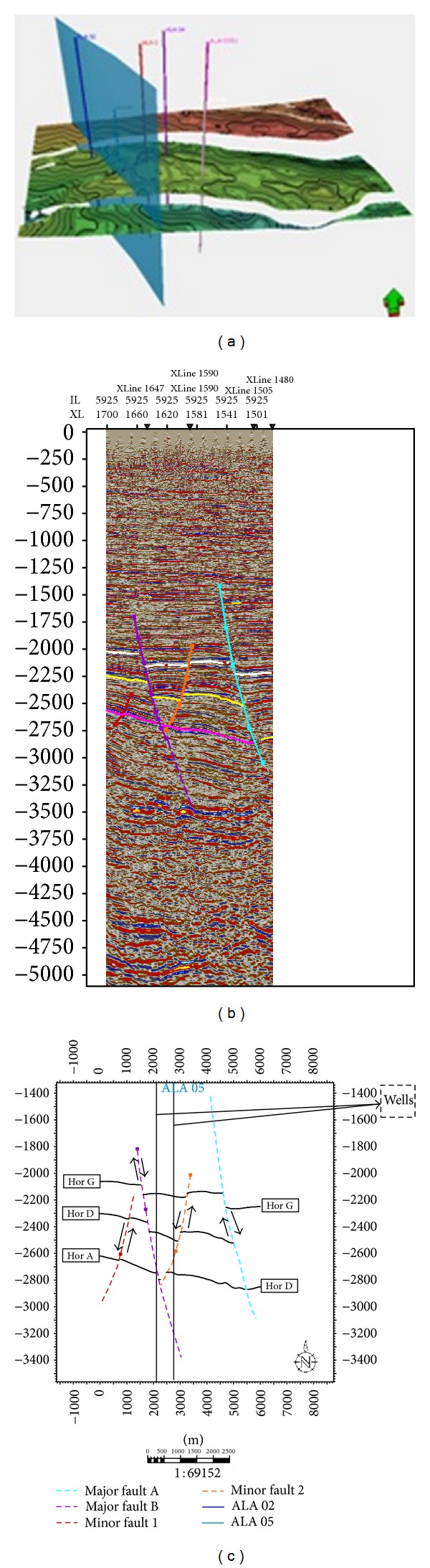
(a) Cross-section path. (b) Seismic section showing the displacement of the reservoir sands. (c) Cross-section showing displacement of the three sands mapped.

**Figure 8:**
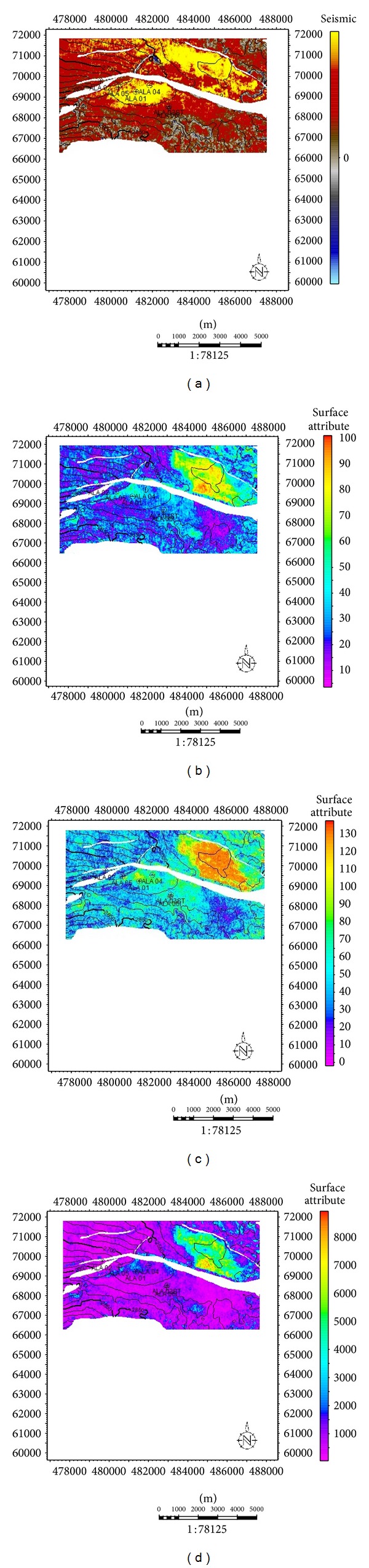
Seismic attribute maps for reservoir A. (a) Acoustic amplitude map for reservoir A. (b) Rms amplitude map for reservoir A. (c) Maximum amplitude map for reservoir A. (d) Average energy for reservoir A.

Sand development in Ala field is somewhat uniform west-east across the field but better developed towards the main structure building fault. Growth faults and anticlines are apparent on this field which serves as traps acting either as fault assisted as in minor fault F2 or anticline closures as in faults F2 and F5 [9]. The anticlines and fault-assisted closures are good hydrocarbon prospects in the Niger Delta [10]. The wells in this field are located on the downthrown block of the major fault M2 (Figures 8(a) and 8(b)), in the rollover anticlines formed against the fault. The capability of the faults to act as seals depends on the amounts of throw and the volume of shale smeared along the fault planes [10]. Faults could act as seals if either the throws are less than 150 m, or the volume of shale smeared along the fault plane is more than 0.25 (25%). Cross-sections (Figure 7) were generated to further comprehend the relationship between the faults and the horizons. The throws of the faults were estimated in this research (Table 1). Poststack seismic attributes as acoustic amplitude, RMS amplitude, maximum amplitude, and average energy which are direct hydrocarbon indicators were generated for the reservoirs mapped to apprehend the geological framework and be able to predict new prospects. From the acoustic amplitude attribute map of reservoir A (Figure 8(a)), two closures—the closure against the fault F2 (closure 1) and that against F5 (closure 2)—are amplitude supported which serve as leads for future drilling project. These areas are bright spots which are indicative of hydrocarbon presence.

Closure 2 which is a prospect area conspicuously has higher amplitude than the present area (closure 1) where all the present wells are drilled (Figure 8). This trend is also noticed on all other amplitude attribute maps generated. On the Rms amplitude attribute map (Figure 8(b)), closure 1 has moderate amplitude ranging from 20 to 53 while closure 2 has amplitude as high as 98. This fact is further buttressed on the maximum amplitude and average energy attribute map (Figure 8(c)) with closure 1 having values range between 80–100 and 1680–4030, respectively, while closure 2 has values as high as 130 and 7985. A third closure (closure 3) situated at the southwestern part of the map is also amplitude supported but not conspicuous; this is most likely not hydrocarbon charged as the high amplitude here could be as a result of high net-to-gross ratio (thick sand deposition). 

**Figure 9:**
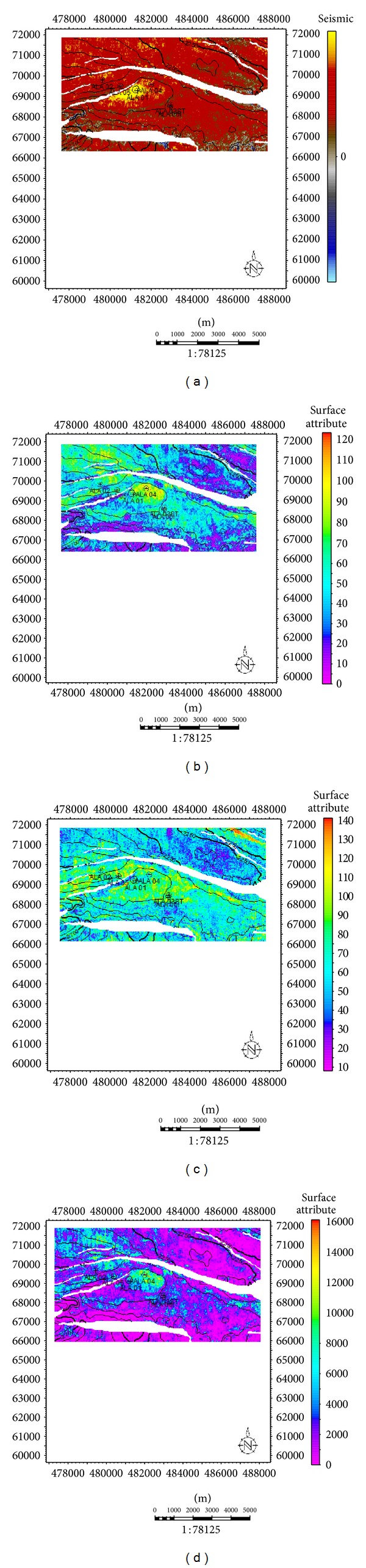
Seismic attribute maps for reservoir D. (a) Acoustic amplitude map for reservoir D. (b) Rms amplitude map for reservoir D. (c) Maximum amplitude map for reservoir D. (d) Average energy for reservoir.

**Figure 10:**
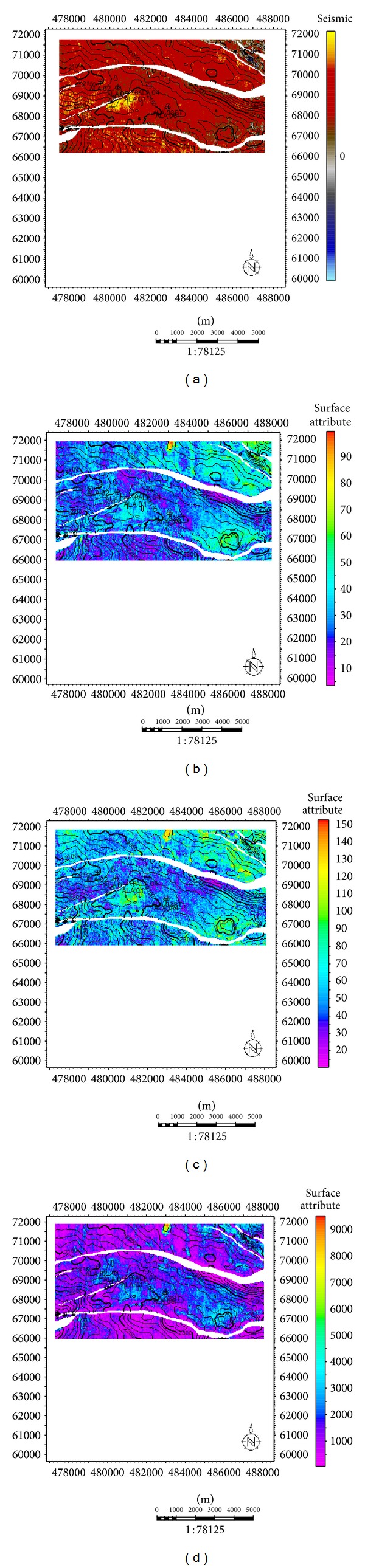
Seismic attribute maps for reservoir G. (a) Acoustic amplitude map for reservoir G. (b) Rms amplitude map for reservoir G. (c) Maximum amplitude map for reservoir G. (d) Average energy for reservoir G.

For reservoir D, the high amplitude is evidenced on closure 1 (Figure 9), which indicates the presence of hydrocarbon. From the Rms amplitude (Figure 9(b)), high amplitude was observed on closure 1, the northwestern area and northeastern edge of the map. The high amplitudes observed on the northwestern area, and northeastern edge of the map most likely would be a result of sand deposition in the area since there is no structure which could support hydrocarbon accumulation; this same pattern is also evident on the maximum amplitude and average energy attribute maps (Figures 9(c) and 9(d)). Interestingly, rms amplitude, maximum amplitude, and energy in closure 1 are as high as 120, 100, and 12400, respectively. From the attribute maps generated for reservoir G (Figure 10), high amplitude on closure 1 is not very conspicuous on all the attribute maps, but it still was fairly distinct from the surrounding environment. This sparse hydrocarbon occurrence was also observed on the well logs, as only ALA 01 showed the presence of hydrocarbon. Most distinct on the attribute maps is the high amplitude on an isolated geologic feature (which is most likely hydrocarbon charged) in the north central part of the maps except for the acoustic amplitude map where low amplitude was depicted. 

**Figure 11:**
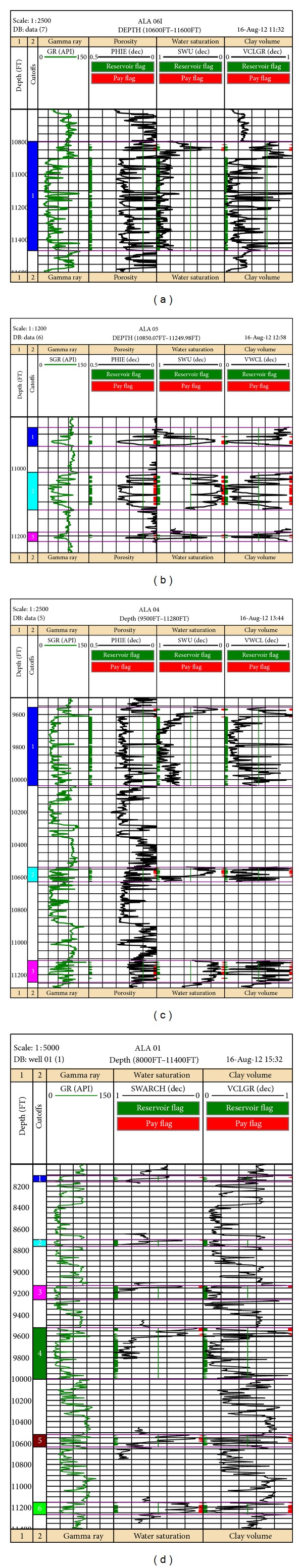
Log plot showing the net pay of the reservoirs in ALA 01, 04, 05, and 06I. (a) Log plot for ALA 06I. (b) Log plot for ALA 05. (c) Log plot for ALA 04. (d) Log plot for ALA 01.

The six wells located within the field penetrated the two upper lithological zones of the Niger Delta. The first zone lies between the depth intervals of 0 ft to −7972.44 ft (−2432 m) and comprised mainly thick sand bodies with few very thin shale interbeds. The second lithological zone extends from the depth of −7972.44 ft (−2432 m) to about −13088.50 ft (−3989.37 m) as shown in Figure 11.

**Figure 12:**
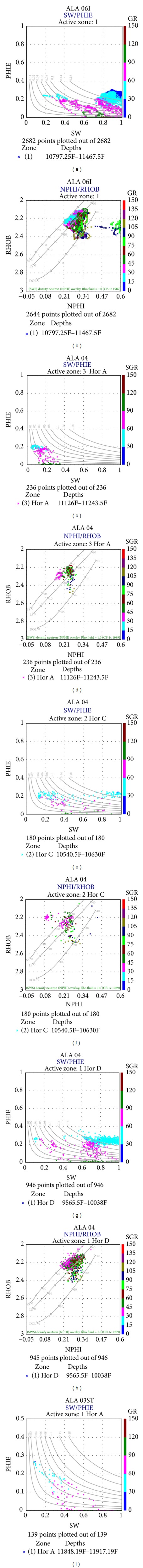
Buckles and neutron-density crossplot for reservoirs in ALA 06I, 04, and 03ST. (a) Buckles plot of reservoir D in ALA 06I. (b) Crossplot of reservoir D in ALA 061. (c) Buckles plot of reservoir A in ALA 04. (d) Crossplot of reservoir A in ALA 04. (e) Buckles plot of reservoir C in ALA 04. (f) Crossplot of reservoir C in ALA 04. (g) Buckles plot of reservoir D in ALA 04. (h) Crossplot of reservoir D in ALA 04. (i) Buckles plot of reservoir A in ALA 03ST.

From the well logs, eight reservoirs were delineated, three of which were mapped across the seismic section. In ALA 06I, only reservoir sand D is hydrocarbon bearing which is evident with the high resistivity in the sand (Figure 11). The reservoir interval is between 10821.5 and 11467.5 ft. The petrophysical parameters derived for this reservoir is shown in Table 2. The pay interval is relatively dirty which is noticeable on the neutron density crossplot with the data points clustering around the sandstone (SS) to limestone (LS) index line (Figure 12(b)). At interval 10830.50 ft, there was a glaring change in the petrophysical parameters, the porosity increased from 16.53% to 21.55% with a decrease in the water saturation and volume of shale from 48.40 to 29.19% and 51.75 to 40.85%, respectively. The buckles plot (Figure 12(a)) reveals that the reservoir is not at irreducible water saturation as the data points do not align along the BVW hyperbolic curve. Three reservoirs (A, B, and H) were delineated in ALA 05. The reservoir intervals are 10881.07–10935.92 ft, and 11012.93–11120.62 ft, 11179.47–11215.97 ft for reservoirs B, A, and H, respectively. Petrophysical parameters were derived for the reservoirs (Table 3). Reservoir A has the highest net pay in all the reservoirs (Figure 11(b) and Table 3). The buckles plot reveals that the reservoirs are not at irreducible water saturation as the data points do not align along the BVW hyperbolic curve except for reservoir A which shows fair alignment.

Three reservoirs (A, C, and D) were delineated in ALA 04. The reservoir intervals are 11126.00–11253.50 ft, 10540.50–10630.00 ft, and 9565.50–10038.00 ft for reservoirs A, C, and D, respectively. Reservoir A has the highest net pay of 49.50 ft and reservoir D the lowest with 15 ft of net pay (Table 3, Figure 11(c)). Petrophysical properties estimated for this well are shown in Table 3 above. The reservoirs as previously noticed in wells 06I and 05 were also not at irreducible water saturation (Figure 12). Neutron density crossplots were used to delineate the lithology of the reservoir sands; this reveals the reservoirs to be shaly sands (Figure 12). In ALA 03ST, only reservoir sand A is hydrocarbon bearing (Table 5) with interval between 11848.194 and 11917.194 ft. The reservoir is very shaly as seen on the neutron density crossplot with the data points clustering around the dolomite trend line; buckles plot (Figure 12(i)) shows the reservoir to be at irreducible water saturation. Petrophysical attributes derived from well logs are shown in Table 4 which reveals a low net-to-gross ratio for this reservoir. In ALA 02, only reservoir sand A is hydrocarbon bearing with interval between 11249.00 and 11346.50 ft. The reservoir has a low net-to-gross ratio in this well (Table 6). Well ALA 01 stands to be the most productive well in this field with six (6) reservoirs (A, C, D, E, F, and G) delineated.

The reservoir intervals are 11152.00–11264.00 ft, 10518.00–10620.00 ft, 9519.00–10002.00 ft, 9131.5–9367.00 ft, 8702.00–8765.50 ft, and 8105.5–8166.00 ft for reservoirs A, C, D, E, F, and G, respectively. Reservoir A has the highest net pay of 71.00 ft and reservoir G the lowest with 7.50 ft of net pay (Table 7 and Figure 11(d)). From the petrophysical analysis, reservoir A is the most laterally continuous and viable.

**Figure 13:**
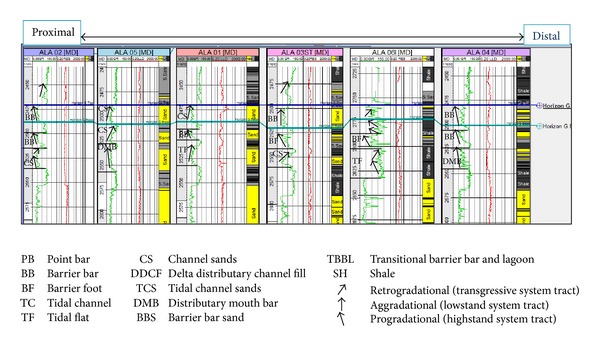
Stratigraphic cross-section of reservoir sand G.

**Figure 14:**
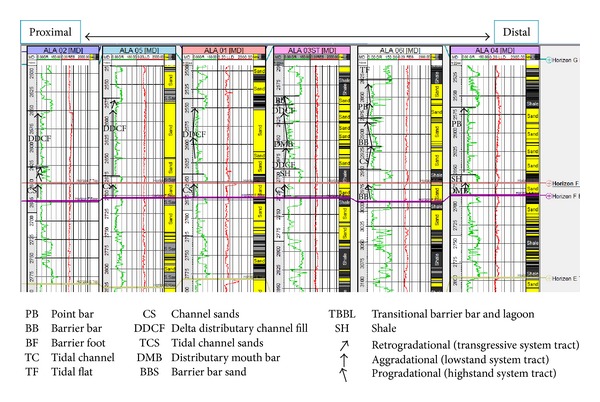
Stratigraphic cross-section of reservoir sand F.

**Figure 15:**
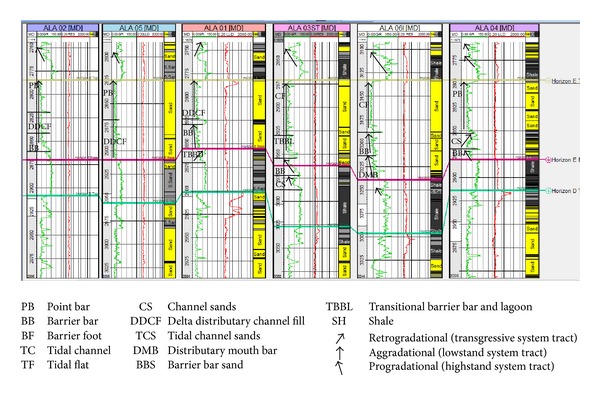
Stratigraphic cross-section of reservoir sand E.

**Figure 16:**
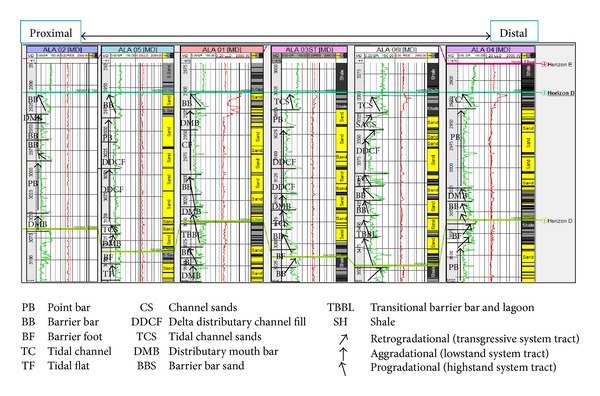
Stratigraphic cross-section of reservoir sand D.

**Figure 17:**
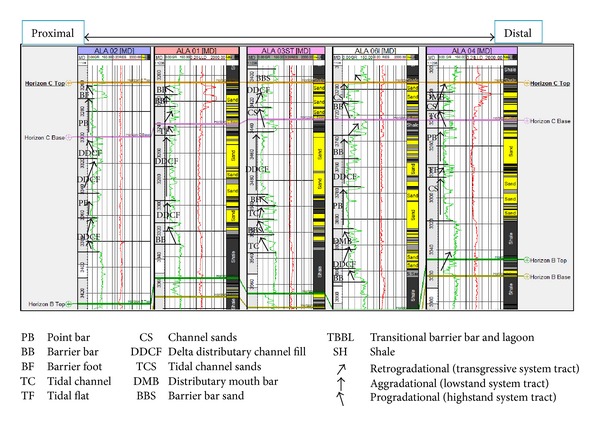
Stratigraphic cross-section of reservoir sand C.

**Figure 18:**
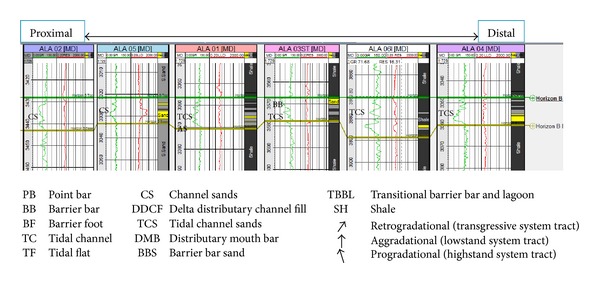
Stratigraphic cross-section of reservoir sand B.

**Figure 19:**
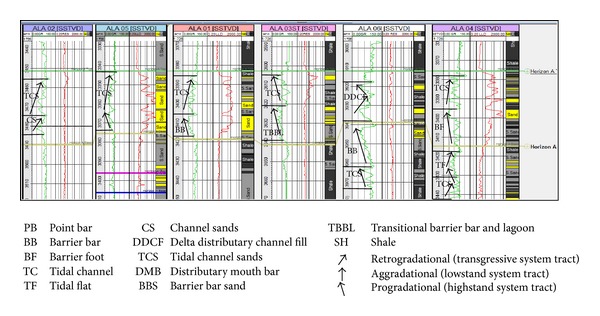
Stratigraphic cross-section of reservoir sand A.

The sandy parts of the paralic sequences (Agbada Formation) are composed of barrier bars, point bars, distributary channels, tidal channels, river-mouth bars, shallow-marine bars, and leeves [10]. Within paralic sequences, reservoir quality is strongly linked to depositional environments. For example, sands of barrier bar origin are more laterally continuous than those of distributary-channel origin. Barrier sands may commonly be correlated along the strike of fields (typically on the order of 10 km), whereas channel sands may be restricted to individual wells [3]. With this in mind, the environment of deposition was inferred from log motifs for the reservoir sands from A to G (Figures 13–19). Field wide log correlations show a wide distribution, both laterally and vertically, of regressive sandstone lithofacies sequences which are predominantly shoreface deposits varying from transitional to deltaic sediments, represented by mudstone and thin sandstone layers, lower to middle shoreface sediments represented by hummocky sandstone deposited under wavestorm influence, upper regressive shoreface sediments represented by coarsening upward sequences or channelized barrier bar complexes with thick sandstone. Reservoir sand A (Figure 19) is generally retrogradational. The sands are tidal channel sands, dominantly sand with relatively thin interbeds of shale dividing the sands into three lobes with varying petrophysical properties. Reservoir sand B (Figure 18) is also tidal channel sand. The sands are turbidities (proximal to distal) and are of high energy environment. Reservoir sand C (Figure 17) has progradational to aggradational pattern and is generally clean. The sands are mostly barrier bars and distributary mouth bars. Reservoir D (Figure 16) is barrier bar to tidal sands. The sands have aggradational to progradational stacking patterns. Reservoir sand E (Figure 15) displays progradational to retrogradational stacking patterns but is generally coarsening upward. The sands are massive with thin shaly interbeds. The depositional environments inferred are point bars and delta distributary channel fills. The sand F (Figure 14) is generally aggradational. The depositional environments eminent are barrier bars, channel sands, and distributary mouth bars. The depositional environment of sand G (Figure 13) varies from barrier bars to channel sands. The underlying sands are tidal flats, barrier foot, distributary mouth bars, and channel sands. The sands are generally progradational. This horizon is immediately below the delineated beginning of Agbada Formation.

## 5. Conclusion

From this research work, it was discovered that reservoir A which is tidal channel sands has the highest net pay; this further buttress the fact that thicker reservoirs in the Niger delta likely represent composite bodies of stacked channels [3].

Most reservoirs encountered on Ala field are point bars, barrier bars, and tidal channel sands and support the work of Kulke [11] which describes the most important reservoir types as point bars of distributary channels and coastal barrier bars intermittently cut by sand-filled channels. Deductions from GR log signatures suggest two (2) regimes of depositional settings within the Ala field. The shallower sands from C to G are very likely to be products of generally prograding, proximal, and delta-front deposits, consisting of shore-face, lower and upper mouth bars and continental shelf deposits; this is buttressed by the high net-to-gross sand ratio observed in these reservoirs while the relatively deeper sands A, B, and H are likely to have been deposited in distal, shallow marine environments which is evidenced by the low net-to-gross sand ratio. These two distinct depositional settings are separated by shale (sand-starved sediments) columns of varying thicknesses. The reservoirs have moderate to high net-to-gross ratios and expectedly have average-good porosity and very high permeability values capable of supporting economic hydrocarbon flow rates. Structurally, the study area is characterized by a distinctively fault-closed dominated structural play. The field structure is an elongate anticline, wedged between the field's west-southeast trending major structure building faults to the north (which is the principal displacement zone) and a northeast-southwest trending fault splay to the south. It can be deduced from this study that the wells drilled on the study area were located to target the rollover anticline formed on the downthrown side of the fault M2 and assisted by minor fault F2.

An amalgamation of the results from petrophysics, seismic structural mapping, and various seismic attributes (acoustic amplitude, rms amplitude, average energy, and maximum amplitude) has revealed closure 2 (the closure against the minor fault F5) as a new prospect where new exploration efforts can be directed to because it is more hydrocarbon charged than closure 1 in the reservoirs. Geochemical and biostratigraphic studies should be employed to better characterize the reservoirs and reliably interpret the depositional environments. Fault seal analysis should also be carried out to check the trapping ability of the major trap which is fault F2. Seismic data for fields bounding this research field should be studied to further understand the nature of the minor fault F4.

## Figures and Tables

**Table 1:** Fault throw estimation.

Fault	Reservoir	Throw (m)	Throw (ft)
Major fault (M1)	G	83.227	273.054
	D	192.150	630.413
	A	—	—

Major fault (M2)	G	54.731	179.564
	D	58.827	193.002
	A	—	—

Minor fault (F1)	G	—	—
	D	15.961	52.365
	A	20.561	67.457

Minor fault (F2)	G	30.906	101.398
	D	99.267	325.679
	A	—	—

**Table 2:** Average petrophysical parameters for ALA 06I.

Sand unit	Net (ft)	Gross (ft)	Φ_*E*_ (%)	*S*_*w*_ (%)	*S*_*H*_ (%)	*V*_sh_ (%)	NGR (%)	*S*_wirr_ (%)	BVW	*V*_hydt_	*V*_rock_	*K* (md)	OWC (ft)
D	22.00	670.25	18.57	35.70	64.30	45.60	80.80	11.47	0.08	0.11	0.41	490.672	10853.80

**Table 3:** Average petrophysical parameters for ALA 05.

Sand unit	Net (ft)	Gross (ft)	Φ_*E*_ (%)	*S*_*w*_ (%)	*S*_*H*_ (%)	*V*_sh_ (%)	NGR (%)	*S*_wirr_ (%)	BVW	*V*_hydt_	*V*_rock_	*K* (md)	OWC (ft)
B	13.00	54.84	20.33	25.30	74.70	22.50	23.70	9.43	0.07	0.14	0.51	1711.620	10928.10
A	66.35	110.36	22.39	11.00	89.00	26.50	60.10	9.56	0.03	0.19	0.64	1575.928	11121.00
H	4.33	27.51	18.46	36.50	63.50	36.50	21.80	12.09	0.07	0.11	0.44	4234.245	11209.00

**Table 4:** Average petrophysical parameters for ALA 04.

Sand unit	Net (ft)	Gross (ft)	Φ_*E*_ (%)	*S*_*w*_ (%)	*S*_*H*_ (%)	*V*_sh_ (%)	NGR (%)	*S*_wirr_ (%)	BVW	*V*_hydt_	*V*_rock_	*K* (md)	OWC (ft)
D	15.00	481.00	24.44	22.10	77.90	36.30	78.30	8.84	0.07	0.18	0.47	2597.534	9590.00
C	36.00	89.50	18.97	25.50	74.50	25.50	70.90	11.35	0.06	0.13	0.49	523.748	10594.50
A	49.50	132.00	19.10	11.20	88.80	25.00	37.50	11.71	0.03	0.17	0.68	5030.835	11249.50

**Table 5:** Average petrophysical parameters for ALA 03ST.

Sand unit	Net (ft)	Gross (ft)	Φ_*E*_ (%)	*S*_*w*_ (%)	*S*_*H*_ (%)	*V*_sh_ (%)	NGR (%)	*S*_wirr_ (%)	BVW	*V*_hydt_	*V*_rock_	*K* (md)	OWC (ft)
A	28.00	69.00	26.22	27.10	72.90	30.30	40.60	8.37	0.08	0.19	0.44	3666.40	11911.70

Net (ft): net thickness, gross (ft): gross thickness, Φ_*E*_ (%): effective porosity, *S*_*w*_ (%): water saturation, *S*_*H*_ (%): hydrocarbon saturation, *V*_sh_ (%): volume of shale, NGR (%): net-to-gross ratio, *S*_wirr_ (%): irreducible water saturation, BVW: bulk volume of water, *V*_hydt_: bulk volume of hydrocarbon, *V*_rock_: matrix volume, *K* (md): permeability, and OWC: oil-water contact.

**Table 6:** Average petrophysical parameters for ALA 02.

Sand unit	Net (ft)	Gross (ft)	Φ_*E*_ (%)	*S*_*w*_ (%)	*S*_*H*_ (%)	*V*_sh_ (%)	NGR (%)	*S*_wirr_ (%)	BVW	*V*_hydt_	*V*_rock_	*K* (md)
A	36.00	97.50	—	—	—	23.20	36.90	—	—	—	—	—

**Table 7:** Average petrophysical parameters for ALA 01.

Sand unit	Net (ft)	Gross (ft)	Φ_*E*_ (%)	*S*_*w*_ (%)	*S*_*H*_ (%)	*V*_sh_ (%)	NGR (%)	*S*_wirr_ (%)	BVW	*V*_hydt_	*V*_rock_	*K* (md)
G	25.25	57.50	—	39.20	60.80	37.70	43.90	—	—	—	—	—
F	12.00	64.50	—	28.50	71.50	16.50	95.70	—	—	—	—	—
E	17.50	134.50	—	29.80	70.20	12.30	98.00	—	—	—	—	—
D	52.00	482.00	—	21.30	78.70	28.20	79.80	—	—	—	—	—
C	37.00	117.00	—	22.00	88.00	17.00	61.80	—	—	—	—	—
A	71.00	114.50	—	15.60	84.40	29.10	62.40	—	—	—	—	—

Net (ft): net thickness, Gross (ft): gross thickness, Φ_*E*_ (%): effective porosity, *S*_*w*_ (%): water saturation, *S*_*H*_ (%): hydrocarbon saturation, *V*_sh_ (%): volume of shale, NGR (%): net-to-gross ratio, *S*_wirr_ (%): irreducible water saturation, BVW: bulk volume of water, *V*_hydt_: bulk volume of hydrocarbon, *V*_rock_: matrix volume, *K* (md): permeability, and OWC: oil-water contact.
